# Genetics of Severe Obesity

**DOI:** 10.1007/s11892-018-1053-x

**Published:** 2018-08-18

**Authors:** Una Fairbrother, Elliot Kidd, Tanya Malagamuwa, Andrew Walley

**Affiliations:** 1 School of Human Sciences, London Metropolitan University, North Campus, 166-220 Holloway Road, London, N7 8DB UK; 2 Institute of Medical and Biomedical Education, St George’s University of London, Cranmer Terrace, Tooting, London, SW17 0RE UK

**Keywords:** Obesity, Leptin, Melanocortin-4 receptor, GWAS, BMI

## Abstract

**Purpose of Review:**

This review aims to present current information on genes underlying severe obesity, with the main emphasis on the three genes *LEP*, *LEPR* and *MC4R*.

**Recent Findings:**

There is a substantial amount of evidence that variants in at least ten different genes are the cause of severe monogenic obesity. The majority of these are involved in the leptin-melanocortin signalling pathway. Due to the frequency of some of the identified variants, it is clear that monogenic variants also make a significant contribution to common obesity.

**Summary:**

The artificial distinction between rare monogenic obesity and common polygenic obesity is now obsolete with the identification of *MC4R* variants of strong effect in the general population.

## Introduction

Obesity is both very common, with a prevalence of 12% globally, and accompanied by high rates of serious, life-threatening, complications such as type 2 diabetes, cardiovascular disease and cancer [1]. Its underlying causes are complex and have proven relatively difficult to elucidate [2•]. A person with a body mass index (BMI) of 30 kg/m^2^, or more, is defined as obese, and severe obesity has been strictly defined as a BMI within the range of 35–39.9 kg/m^2^ [3]. However, severe obesity is frequently defined with the broader meaning of having a BMI of greater than 35 kg/m^2^, that is it includes obesity classes II, III and IV (see Table 1). BMI has been used to assess obesity rates in populations in relation to health with some considerable success [4], and prevalence is high in many countries, such as the USA and UK (see Table 1). The economic cost of obesity to national health systems and the wider society is a considerable burden on wealthy economies [5], particularly severe obesity [6] and an alarming prospect for such emerging large economies as China where obesity rates are rising fast [7].

**Table 1:** Body mass index (BMI) ranges, corresponding descriptions and rates of each category in the USA and UK for adults of both sexes in the latest year for which data was available*

BMI range (kg/m^2^)	Description	USA %	UK %
< 18.5	Underweight	2.4	5.1
18.5–24.9	Normal weight	35.7	33.9
25–29.9	Overweight	32.2	38.3
30–34.9	Obesity class I (obese)	33.9	22.70
35–39.9	Obesity class II (severely obese)		
> 40	Obesity class III (morbidly obese		
> 50	Obesity class III (super obese)		

Table created using data from WHO Global Health Observatory data; http://www.who.int/gho/ncd/risk_factors/overweight/en/

While it has been evident for many years that there is a strong genetic component affecting obesity, with twin studies providing reproducible heritability values as high as 0.77 across different regions of the world and at different ages [8•], there has been relatively little investigation of severe obesity specifically. This is almost certainly due to the considerable overlap between rare monogenic obesity and common polygenic obesity at this part of the distribution of BMI within the population. However, at least one study has attempted to investigate genetic effects in severe obesity [9]. This used a definition of morbid obesity of 45.5 kg over the ideal weight of an individual, something that would actually place many people in the category of severely obese based on current definitions (see Table 1). Family members of probands were found to be eight times more likely to be severely obese than the general population. They also demonstrated that families with at least one severely obese parent were 2.6 times more likely to have one or more severely obese adult offspring compared to the general population. This suggested that genetic effects were important in this sub-group of the obese but could not eliminate the effects of shared environment.

With a role for genetics well established in obesity, the focus for this review is on severe obesity as a distinct disease with a specific genetic cause rather than syndromic or common polygenic obesity. The overlap of rare monogenic disease with common obesity will also be discussed.

## Monogenic Severe Obesity

This review will concentrate on the original obesity gene, leptin (*LEP*) and its receptor (*LEPR*), together with a gene that has been identified as having variants of strong effect giving rise to both monogenic obesity and obesity in the general population, the melanocortin-4 receptor (*MC4R*). Additional selected genes reported to be associated with severe obesity are listed in Table 2, to provide a flavour of the current state of the art without attempting to be comprehensive.

**Table 2:** Selected genes with variants contributing to severe monogenic obesity with recent reviews

Gene symbol	Full name	Location	Obesity	Associated traits	Recent reviews
*ADCY3*	Adenylate cyclase 3	2p23.3	Severe-early onset	Type 2 diabetes	Tian et al. 2018 [10]
*BDNF*	Brain-derived neurotrophic factor	11p14.1	Severe-early onset	Hyperphagia, severe obesity, hyperactivity and impaired cognitive function.	Han et al. 2016 [11]
*KSR2*	Kinase suppressor of ras 2	12q24.22-q24.23	Severe	Hyperphagia (in childhood), insulin resistance and reduced basal metabolic rate.	Frodyma et al. 2017 [12]
*LEP*	Leptin	7q32.1	Severe, early onset	Hyperphagia, hypogonadotropic hypogonadism. Some evidence for neuroendocrine/metabolic and immune dysfunction	Wasim et al 2016; Yeo 2017 [13, 14]
*LEPR*	Leptin receptor	1p31.3	Severe, early onset	Hyperphagia, hypogonadotropic hypogonadism. Some evidence for neuroendocrine/metabolic and immune dysfunction	Wasim et al 2016; Yeo 2017 [13, 14]
*MC4R*	Melanocortin 4 receptor	18q21.32	Severe, early onset	Increased linear growth and final height, fasting hyperinsulinemia and incompletely suppressed growth hormone secretion.	Krashes et al 2016; Yeo 2017; Novoselova et al 2018 [14–16]
*PCSK1*	Proprotein convertase subtilisin/kexin type 1	5q15	Severe – occurring in childhood	Hyperphagia, impaired glucose homeostasis, decreased linear growth, hypothyroidism, hypocortisolism and hypogonadotropic hypogonadism	Ramos-Molina et al 2016; Stijnen et al 2016 [17, 18]
*POMC*	Proopiomelanocortin	2p23.3	Severe, from the first months	Adrenocorticotropic hormone (ACTH) deficiency, red hair and pale skin	Anderson et al 2016; Rubinstein and Low 2017 [19, 20]

Early work with mouse models implicated leptin and its cognate receptor as implicated in monogenic obesity [21, 22]. Rare human cases where leptin, or the leptin receptor, is entirely ablated have subsequently been reported and uniformly exhibit severe obesity [23–25].

## Leptin

Leptin is a hormone secreted by adipose tissue [26] and leptin levels are directly related to adiposity in humans [27]. It is constitutively expressed, and in conditions of prolonged caloric deficit, fat stores and leptin production will decrease [28]. It is a cytokine (or adipokine) essential for regulation of energy balance through feeding behaviour and energy expenditure [21]. Leptin is anorexigenic and seems likely to be our main adiposity indicator and signal of nutritional status: plasma levels are highly correlated to adipocyte number and fat mass [29]. Leptin levels are also strongly correlated with insulin resistance independently of fat volume; thus, hyperleptinaemia can be considered an independent factor in obesity (see [30] for review).

Leptin signals by binding to the leptin receptor (a type I cytokine receptor) in the arcuate nucleus of the hypothalamus, reducing the desire to eat and stimulating thermogenesis [31]. Leptin signalling is via the JAK-STAT pathway [32–34].

Circulating leptin binds to the soluble form of the leptin receptor, sOB-R [35], and activates Janus kinase (JAK2), which then phosphorylates three tyrosine residues in LEPR*,* which then induces the phosphorylation of STAT transcription factors STAT5 and STAT3 (see [36] for review). This provides the metabolic link between leptin levels and the many downstream energy homeostatic pathways it regulates, which include growth, caloric expenditure and glycaemic control. In addition to behavioural control regarding food intake and secretion of adrenal corticosteroids, circulating leptin induces its effects via LepRb [37], the long form of its receptor, which is expressed specifically in areas of the brain with a role in feeding and energy expenditure [38]. In normal weight individuals, if leptin is decreased due to reduction in white adipose tissue, such as during prolonged starvation, this induces orexigenic signalling, which results in decreased energy use and disruption of glucose homeostasis (see [38] for review).

The mechanism by which this pathway is dysregulated is complex and has been demonstrated to include factors that have varying levels of influence such as feedback inhibition, inflammatory responses, gliosis and endoplasmic reticulum stress [39]. Circulating leptin levels are increased in obese humans and also in animal models, but the essential feedback mechanism that promotes reduction in feeding behaviour and increased energy expenditure fails [40]. Interestingly, leptin receptors continue to respond to the increased leptin [41]. It appears that an obese end point is reached despite increased leptin-leptin receptor signalling but that continuous high levels of leptin receptor signalling induce leptin resistance and “caps” the amplitude of the signal [39, 42].

There are currently only eight different mutations reported in the *LEP* gene that are thought to cause severe obesity (see [13] for review). Recent studies, including exome sequencing, have increased the numbers of known variants but *LEP* mutations remain very rare (see Table 2). Allelic variation has been extensively studied with the view that these may confer greater propensity to obesity. For example, family-based association analysis of a large consanguineous Tunisian family identified the functional variants (H1328084 and A19G) in the 5′ UTR of *LEP.* These variants have been associated with plasma leptin level as a quantitative trait and are thus implicated in affecting plasma leptin levels [43].

Many candidate gene and genome-wide association studies (GWAS) have been carried out where *LEP* and *LEPR* polymorphisms have been investigated for their role in measures of adiposity, obesity and its sequelae [13, 44–48]. There is a relatively small amount of literature positively implicating *LEP* polymorphisms in this role and the relationship is dependent on ethnicity and age of subject [44, 49]. *LEP* G2548A has been associated with obesity and serum leptin levels in Turkish subjects [50] and a study that reports a synergistic effect of *LEP* and *LEPR* polymorphisms on BMI, in a Han Chinese population [51]. In a Pakistani population, this polymorphism has also been shown to be associated with obesity in female children < 18 years old. In addition, G-2548A polymorphism showed association with BMI, fasting blood glucose and serum leptin levels in male and female children [52].

It is clear that ascertainment of traits has a profound impact on identification of positive associations. *LEP* polymorphisms G2548A in a recent meta-analysis involving 1372 obese individuals (BMI > 30 kg/m^2^) and 1616 controls concluded that there was no association with *LEP* [49]. However, three studies show a positive association with severe obesity in Taiwanese aboriginals and Caucasians [53–55].

## Leptin Receptor

The leptin receptor gene, *LEPR* at 1p31, encodes a single membrane spanning receptor of the class I cytokine receptor family [22]. There are six leptin receptor or ObR isoforms produced by alternative splicing [56, 57]. With different isoforms being expressed in different tissues [58], these include one long form (LEPR or ObRb), four short forms (ObRa, ObRc, ObRd and ObRf, with unique C-termini) and one soluble form (ObRe). LEPR, the long form, which is the isoform truncated in obese mice (ob/ob) and known to be important in energy and feeding control, contains three highly conserved tyrosine residues (Y985, Y1077, Y1138) required for efficient leptin signalling [21, 38, 59]. Its major site of expression is the arcuate nucleus neurones, which are known to have a role in energy homeostasis and are reported to be responsive to leptin signalling (see [60] for review). Many of these neurones also express pro-opiomelanocortin (POMC) in response to excitation by leptin, and via central nervous system (CNS) melanocortin receptors (MCRs), play a key role in negatively moderating feeding behaviour and increasing energy expenditure [38, 61].

Truncation of LEPR has been shown to cause morbid monogenic obesity and severe hyperphagia in mice (*db/db*) and humans [37, 62–64]. Genome scans and association studies have linked LEPR to measures of adiposity including energy expenditure in: a population with elevated obesity levels, fat mass, skinfold and fat-free mass; BMI trends in childhood; and leptin levels, body composition, insulin dysregulation and glucose metabolism. The leptin receptor also has a role in the hypothalamic-pituitary-gonadal axis, thus influencing onset of puberty [65].

Numerous coding single-nucleotide variants (SNVs) have been identified in the *LEPR* gene including: Lys109Arg (rs1137100), which lies in the cytokine homology domain (CK); Gln223Arg (rs8179183) in the loop region of the CK domain; and the Lys656Asn (rs8179183), in the fibronectin type III (F3) domain [66, 67]*.* These domains are common to all the isoforms.

The association of these variants with common severe obesity and obesity-related phenotypes has been reinforced through evidence generated by a multitude of candidate gene studies [44, 48]. Understanding common complex obesity through identification of genes mutated in rare severe obesity must be carefully interpreted in terms of the direction of the effects. In addition, care must be taken in selecting a sample group that is not phenotypically heterogeneous, i.e. overweight, obese and morbidly obese pooled, for example. Indeed, extreme phenotyping is a refinement of this practice and has had some notable successes in identifying genes implicated in quantitative traits, including obesity and obstructive sleep apnoea, for which obesity and leptin levels are significant risk factors [68, 69]. With complex disease, subtle phenotypic heterogeneity may mask underlying contributions from variants of modest effect, especially since the precise phenotypic effect of the variant is unknown and may be different in different ethnic groups. Nevertheless, there is overwhelming evidence that common variants in *LEPR* are associated with measures of adiposity. This seems unlikely to be a direct effect but is likely to be due to subtle variations in functionality whose effect is amplified in downstream targets [39].

## Melanocortin-4 Receptor

The *MC4R* protein is a membrane-bound G-protein-coupled receptor found in several brain regions, including the paraventricular nucleus (PVN) in the hypothalamus (see [70] for review). Stimulation of LEPR on POMC neurons causes them to release α-melanocyte-stimulating hormone (α-MSH) (see [38] for review), which binds to the MC4R protein. This results in the exocytosis of brain-derived neurotrophic factor (BDNF) and neurotrophic tyrosine kinase receptor 2 (NTRK2) (see [71] for review), which are both anorexigenic signals. It should be noted that very recent studies have reported that leptin only directly regulates Agouti-related protein (AGRP) neurons [72, 73]. With many functional relationships regulating the orexigenic-anorexigenic signal balance, it is not surprising that variation in the genes involved in the melanocortin-leptin pathway can give rise to severe obesity [70, 74].

## *MC4R* Gene Sequence Variation

*MC4R*-deficient patients are affected by hyperphagia and, consequently, a higher caloric intake [75, 76]. *MC4R* variation was recognised relatively early as a monogenic cause of severe obesity, accounting for as much as 6% of people with early onset obesity [77]. Most people affected are heterozygous, demonstrating autosomal dominant inheritance [78, 79]. Homozygous cases have also been reported and they display a more severe form of obesity [77, 80].

Currently, there are 376 SNVs and 189 copy number variants reported in the *MC4R* gene region. A total of 182 of the SNVs are missense, 10 are nonsense and 12 are frameshift variants. Of these SNVs, 69 are predicted pathogenic, or likely pathogenic. Only two SNVs have been identified with a minor allele frequency (MAF) > 1% in the 1000 genomes (1000G) or the ExAC reference population datasets: namely rs34114122 (1000G MAF = 6.0%, ExAC MAF = not available) and rs2229616 (1000G MAF = 1.6% and ExAC MAF 1.7%) (data from NCBI Variation Viewer, 27th June 2018) [81, 82].

## Clinical Phenotype

Obese patients with mutant *MC4R* genes are very similar to other obese patients with no identified *MC4R* mutation. They share a similar mean BMI with other obese patients, as well as maximum BMI reached during adult life and minimal BMI reached during caloric restriction [80]. No difference in food intake, incidence of diabetes and glucose intolerance has been observed between the two groups. Fasting glucose, triglyceride levels and mean leptin levels were also the same. However, when looking at childhood obesity, *MC4R* mutant carriers have a higher percentage body fat composition of 67.0 vs 45.5% in other obese children.

When comparing BMI standard deviation, individuals with heterozygous mutations had a score of 2.79 ± 1.61 (mean ± SD) while homozygotes for *MC4R* mutations had a score of 4.81 ± 1.63 (mean ± SD). During the first 5 years of life, mutant *MC4R* carriers show a higher standard deviation for height, a higher body fat percentage of 42.9% compared to 15–25% normal body fat percentage range and a higher fat-free mass than homozygous wild-type subjects, suggesting that MC4R deficiency is characterised by increased fat and lean mass. Therefore, children with MC4R deficiency were taller and more obese than their peers. The study also proved that these children have a higher bone mineral density which corresponds with previous studies [77].

As these patients grow older, hyperphagia decreases and their metabolic rate becomes similar to healthy individuals. Adult *MC4R* mutation carriers do not have an increased risk of diabetes and the hyperinsulinemia seen in children decreases to normal levels after the age of 10. These individuals also show normal endocrine function. There is no strong evidence that binge eating is a phenotype of MC4R deficiency as originally suggested [83–85].

Bariatric surgery is currently the only successful option in treating obesity, but when bariatric surgery has been performed on homozygous mutant *MC4R* patients, it had no impact on long-term weight loss [86, 87].

## GWAS for Severe Obesity

The identification of genes underlying common obesity has been predominantly focused on the phenotype of BMI. This was due to three reasons: the presence of this phenotype in many cohorts recruited for other reasons, the clear relationship with definitions of obesity in populations and the fact it is a simple to measure quantitative trait. However, the first association reported with common obesity originated from a type 2 diabetes study. Variants in the *FTO* gene were associated with type 2 diabetes at genome-wide significance, but this association was not significant when BMI was taken into account [88]. This has led to very many GWAS and meta-analyses for BMI conducted with hundreds of thousands of subjects (e.g. [89, 90]). The main problem with the focus on BMI is that the associations detected are to variants that contribute to the distribution of BMI within a cohort, rather than the variants that associate with obesity specifically.

There are fewer GWAS that have investigated associations with severe obesity and those that have are relatively small and under-powered, with the exception of the most recent meta-analysis (see below). One of the earliest GWAS used both adults with morbid obesity and obese children, arguing that early-onset obesity was likely to be predominantly genetic. Associations were reported with the two genes already identified in the general population, *FTO* and *MC4R*, but associations were also reported with SNVs in the *NPC1* gene and near to the *MAF* and *PTER* genes [91]. Recently, it has been reported that rare variants in the *NPC1* gene are enriched in young severely obese Chinese subjects and that heterozygosity for these variants also leads to increased BMI compared to age-matched controls [92]. The variant near to the MAF gene is midway between the *MAF* gene and the *MAFTRR* gene (*MAF* transcriptional regulator RNA), a long non-coding RNA that regulates MAF expression, suggesting that the association may be with a causal variant that affects transcription of *MAF*, or another, as yet undiscovered, target of MAFTRR. The variant reported near to the *PTER* gene is in fact closer to several non-coding RNA genes, including the long non-coding RNA *RP11-461K13.1* and the U6 small nuclear pseudogene *RNU6-1075P*, again suggesting the possibility that the association is in fact to a variant that affects expression of gene targets of these non-coding RNAs.

A stepwise analytical approach was used in another study where an initial small GWAS was carried out in 164 morbidly obese and 163 always-lean adults, followed by taking the positive associations in two further stages of 700 SNVs in 460/247 cases and controls and then 23 SNVs in 4214 obese versus 5417 lean or population-based controls [93]. The initial GWAS demonstrated nominal association (*p* < 0.05) with variants in brain-derived neurotrophic factor (*BDNF*) and *MC4R*, but not in *FTO*. Variants in the genes *KCNMA1* (potassium calcium-activated channel subfamily M alpha 1) and *BDNF* were reported to be associated with obesity at genome-wide significance. Notably, the SNV in *KCNMA1*, rs2116830, was not associated with BMI in the population-based controls and the *KCNMA1* transcript was over-expressed in adipose tissue in obese adults. This suggests the possibility that *KCNMA1* is purely associated with obesity rather than BMI, but given the relatively small numbers of controls, this result might simply reflect a lack of statistical power.

A GWAS for severe early-onset obesity in a total of 2480 children reported four loci, namely leptin receptor (*LEPR*), protein kinase C eta (*PRKCH*), phosphofurin acidic cluster sorting protein 1 (*PACS1*) and rhabdomyosarcoma 2-associated transcript (*RMST*) [94]. Association of the previously reported 43-kb deletion near to *NEGR1* was also reported but this was determined to be due to linkage disequilibrium with an 8-kb deletion the other side of the *NEGR1* locus. Pathway analysis was reported to suggest enrichment of g protein coupled receptors involved in the neuronal regulation of energy homeostasis. It is notable that similar to the earlier GWAS, association was seen to a locus containing a long non-coding RNA, *RMST*, suggesting that non-coding RNA loci deserve more attention as containing possible obesity-causing variants.

More recently, a very large meta-analysis of the data available to the GIANT consortium generated seven new loci associated with BMI, two of which were associated with class II severe obesity [95••]. The study analysed 15,334 cases and 97,858 controls for the specific class II analysis and identified two novel loci, as well as previously reported associations with *MC4R* and *KCNMA1*. The new loci were *HS6ST3* (heparan sulfate 6-O-sulfotransferase 3) and *ZZZ3* (zinc finger ZZ-type containing 3). Neither of the new genes identified have any reported functional relationship with obesity or BMI.

Study sizes continue to grow in order to increase statistical power, in response to the challenge of detecting loci of small effect, with MAF values < 0.05. Recently, in a study analysing data from > 700,000 subjects on an exome array, a further eight novel gene loci have been associated with BMI, implicating novel candidate pathways involved in obesity and related phenotypes [96•]. The strength of this study is that the cohort is over twice the size of most other GWAS studies to date. Interestingly, these data suggest that *MC4R* and *KSR2* were identified as having a role in common, complex obesity. These genes have previously been identified in much smaller studies, where subjects’ obese phenotype was severe and early onset [69, 89, 97, 98].

Two *MCR4* SNVs were used in the analysis that were array-wide significant at *p* < 2 × 10^−7^: a missense mutation (Asp37Val, rs12447325) and a nonsense mutation (Tyr35Ter, rs13447324), both with a MAF of 0.01%, thus present in 1/5000 subjects. The Tyr35Ter mutation which leads to MC4R deficiency and hyperphagia [75, 76, 78] results in approximately 7 kg of extra weight for a person 1.7 m in height and gave the largest effect size in the whole study. Interestingly, *MC4R* Ille251Leu (rs52820871) is reported in the literature as ameliorating obesity [99]. The *KSR2* variant identified in this study (Arg554Gln, rs56214831) increases body weight by 0.74 kg/allele and is known to be associated with hyperphagia-induced obesity, low basal metabolic rate and insulin resistance in mice and humans [69, 100–102].

Effect sizes of rare variants are generally higher than common SNVs positively identified in GWAS and this is the case for *MC4R* and *KSR2*, but penetrance is low and the 14 SNVs, identified in this large exome array study [96•], combined represent < 0.1% of BMI variation. Of the new loci identified, *EPAC1* (*RAPGEF3*) is known to play a role in energy homeostasis, diabetes and obesity propensity and regulates insulin and leptin signalling [103–105]. Interestingly, knockdown models of adipose specific *epac* were lethal in *Drosophila*, which was not the case for the eight other newly identified loci investigated [96•].

## Conclusions

Over 20 years since the report of the first gene involved in obesity, we now have a far better understanding of the genetics of severe obesity. We have identified a range of genes responsible for severe monogenic obesity and we now know that some of these are frequent enough to be significant causes of obesity in the general population. The boundary between rare monogenic obesity and common polygenic obesity is now becoming blurred. This is not unexpected but it has not really become evident until recently with the technological advances in genotyping and sequencing that now allow us to characterise all variants across the whole genome in large numbers of cases and controls.
